# Pathogenic mutation hotspots in protein kinase domain structure

**DOI:** 10.1002/pro.4750

**Published:** 2023-09-01

**Authors:** Kirill E. Medvedev, R. Dustin Schaeffer, Jimin Pei, Nick V. Grishin

**Affiliations:** ^1^ Department of Biophysics University of Texas Southwestern Medical Center Dallas Texas USA; ^2^ Eugene McDermott Center for Human Growth and Development University of Texas Southwestern Medical Center Dallas Texas USA; ^3^ Department of Biochemistry University of Texas Southwestern Medical Center Dallas Texas USA

**Keywords:** domain, kinases, mutation hotspot, protein structure

## Abstract

Control of eukaryotic cellular function is heavily reliant on the phosphorylation of proteins at specific amino acid residues, such as serine, threonine, tyrosine, and histidine. Protein kinases that are responsible for this process comprise one of the largest families of evolutionarily related proteins. Dysregulation of protein kinase signaling pathways is a frequent cause of a large variety of human diseases including cancer, autoimmune, neurodegenerative, and cardiovascular disorders. In this study, we mapped all pathogenic mutations in 497 human protein kinase domains from the ClinVar database to the reference structure of Aurora kinase A (AURKA) and grouped them by the relevance to the disease type. Our study revealed that the majority of mutation hotspots associated with cancer are situated within the catalytic and activation loops of the kinase domain, whereas non‐cancer‐related hotspots tend to be located outside of these regions. Additionally, we identified a hotspot at residue R371 of the AURKA structure that has the highest number of exclusively non‐cancer‐related pathogenic mutations (21) and has not been previously discussed.

## 1 INTRODUCTION

Post‐translational modification of proteins through phosphorylation is a common process in biology. Protein kinases play an essential role in cellular signaling and regulation and are responsible for phosphorylation. There are more than 500 human protein kinases, which are grouped into several families based on their structure and function and which are encoded by ∼2% of all human genes (Manning et al., 2002). Major kinase groups from the human kinome include AGC (intracellular signaling kinases), CMGC (responsible for cell cycle control, MAPK signaling, splicing, etc.), CAMK (Calmodulin/Calcium regulated kinases), CK1 (Cell Kinase 1, originally known as Casein Kinase 1), STE (form the MAPK cascade), TK (Tyrosine kinases), TKL (similar to TK, but whose activities are generally on serine/threonine substrates), and RGC (Receptor Guanylate Cyclases) (Manning et al., 2002). Protein kinases are involved in many cellular pathways, and their dysregulation has been implicated in various diseases, including cancer, diabetes, neurodegenerative and cardiovascular disorders, and rheumatoid arthritis, among others (Lahiry et al., 2010; Lee & Paull, 2021). Being one of the largest families of evolutionarily related proteins, kinases phosphorylate particular amino acids of 30% of the human proteome (Hubbard & Cohen, 1993) and therefore are one of the most important group of drug targets together with G‐protein‐coupled receptors (Cohen, 2002; Melnikova & Golden, 2004). Kinase inhibitors, which block the activity of specific kinases, have been developed to treat various types of cancer, autoimmune diseases, and other disorders (Melnikova & Golden, 2004). The role of kinases in carcinogenesis has been intensively studied: the protein kinase domain is the most frequently encoded domain among cancer‐related genes (Futreal et al., 2004). In normal cells kinases function as both tumor suppressors and proto‐oncogenes, therefore mutations in these proteins facilitate oncogenic processes such as inhibition of DNA damage response, promotion of angiogenesis, deactivation of apoptotic pathways, and others (Torkamani et al., 2009).

**FIGURE 1:**
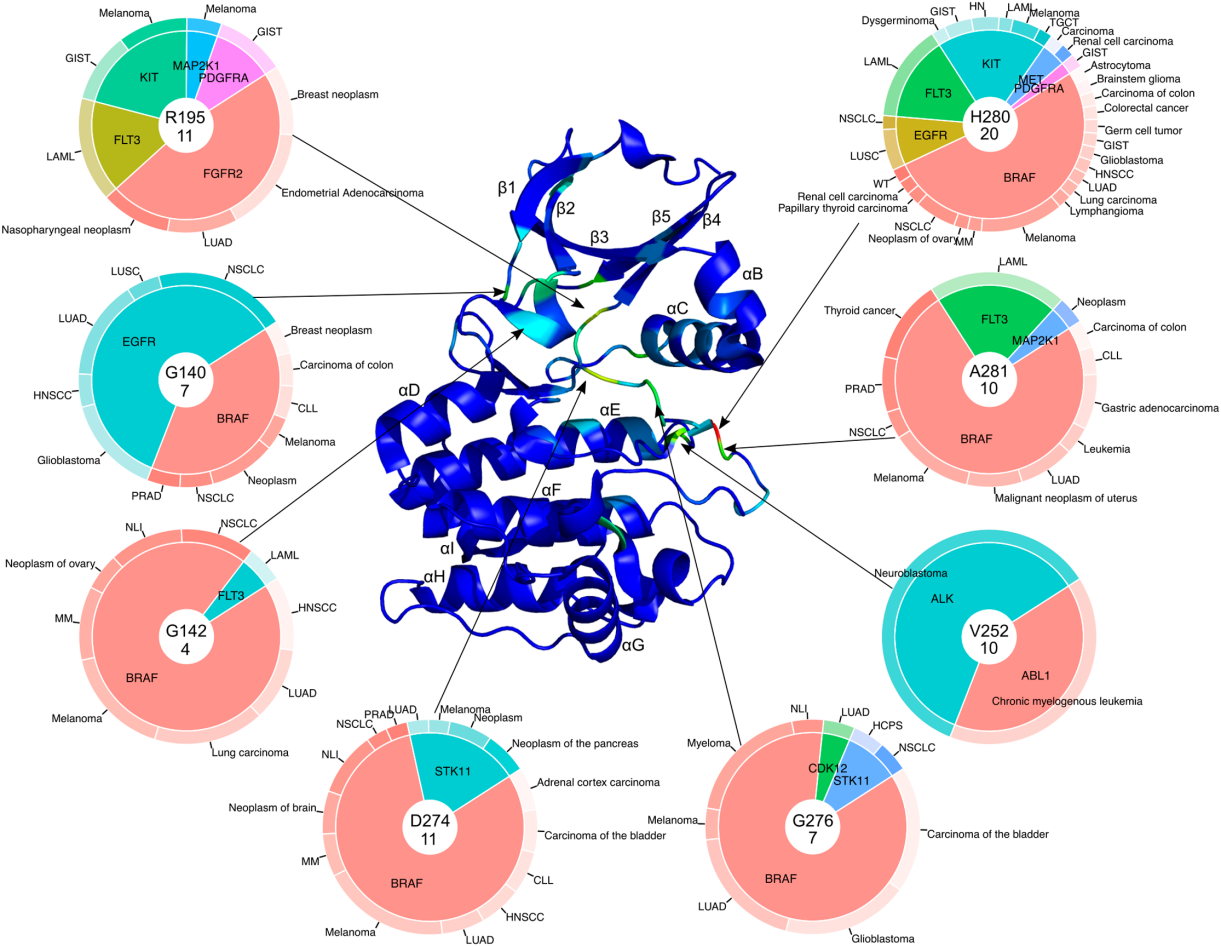
Cancer‐related mutations mapped to kinase domain of Aurora kinase A (PDB: 3E5A). Pie charts represent genes (inner layer) and medical conditions and diseases (outer layer) that are related to mutations in a particular position. One mutation can be related to several diseases. In the center of each pie chart the top item represents the number of amino acid in 3E5A structure, the bottom item represents the total number of SNVs in this position in MSA. CLL, B‐cell chronic lymphocytic leukemia; GIST, gastrointestinal stromal tumor; HCPS, hereditary cancer‐predisposing syndrome; HN, hematologic neoplasm; HNSCC, squamous cell carcinoma of the head and neck; LAML, acute myeloid leukemia; LUAD, lung adenocarcinoma; LUSC, squamous cell lung carcinoma; MM, mltiple myeloma; NLI, neoplasm of the large intestine; NSCLC, non‐small cell lung carcinoma; PRAD, prostate adenocarcinoma; TGCT, germ cell tumor of testis; WT, nephroblastoma (Wilms tumor).

The secondary structure of a kinase domain includes two structurally and functionally distinct subdomains or lobes: N‐ and C‐terminal (Taylor & Kornev, 2011). The smaller N‐terminal lobe adopts a five‐stranded β‐sheet and includes highly conserved sequence motif—Gly‐rich loop between β1 and β2. This loop interacts with adenine ring of ATP (Madhusudan et al., 2002). The AGC group of typical protein kinases shares the distinctive helix αB in the N‐terminal region (Figure 1). This helix is not observed in any of the other type of typical kinases (Scheeff & Bourne, 2005). The majority of kinases typically exhibit a noticeable kink near the beginning of the strand β4. This kink alters the arrangement and structure of a significant portion of the hydrophobic pocket formed by strand β4, helices αC, and αE (Scheeff & Bourne, 2005). The larger C‐terminal lobe contains mostly alpha helices. This lobe also includes the catalytic loop that bridges β6 and β7 and activation loop right after the catalytic region (Taylor & Kornev, 2011). The catalytic region of many kinase families is reinforced by intricate hydrogen‐bond networks that stabilize the structure of the active site. Moreover, within this region, a distinctive “crossing loops” structure is formed, where the catalytic loop intersects with the loop between strands β8 and β9. This motif is uncommon in protein structures and serves as one of the distinguishing features of the kinase superfamily (Grishin, 1999). Helix αE plays a stabilizing role in the ATP binding pocket through its interactions with strands β7 and β8. In the majority of kinases, αE is oriented at approximately 45° to these strand elements (Scheeff & Bourne, 2005). Helix αF, following the diverse loop structures, represents the final segment of structural similarity shared by all kinases. However, the similarity in this region diminishes rapidly. In certain instances, it could be argued that this helix exhibits poor superposition among superfamily structures and that it should not be regarded as part of the “universal core” (Scheeff & Bourne, 2005). However, αF consistently appears with a similar orientation in most of the kinases' structures, and in many cases, seems to serve a similar role: the stabilization of the backbone of the catalytic loop.

Here we identified pathogenic mutation hotspots using data from the ClinVar database (Landrum et al., 2020) and an alignment of 497 human protein kinase domains. We defined hotspot mutations based on the frequency of mutations among kinase domains. Our analysis showed that most of the cancer‐related mutation hotspots are located within catalytic and activation loops of kinase domain, whereas most of non‐cancer‐related hotspots are mainly located outside of these loops. We also identified the hotspot with the highest number of exclusively non‐cancer‐related pathogenic mutations (21) that corresponds to R371 of the AURKA structure and has not been discussed previously.

## 2 RESULTS AND DISCUSSION

**FIGURE 2:**
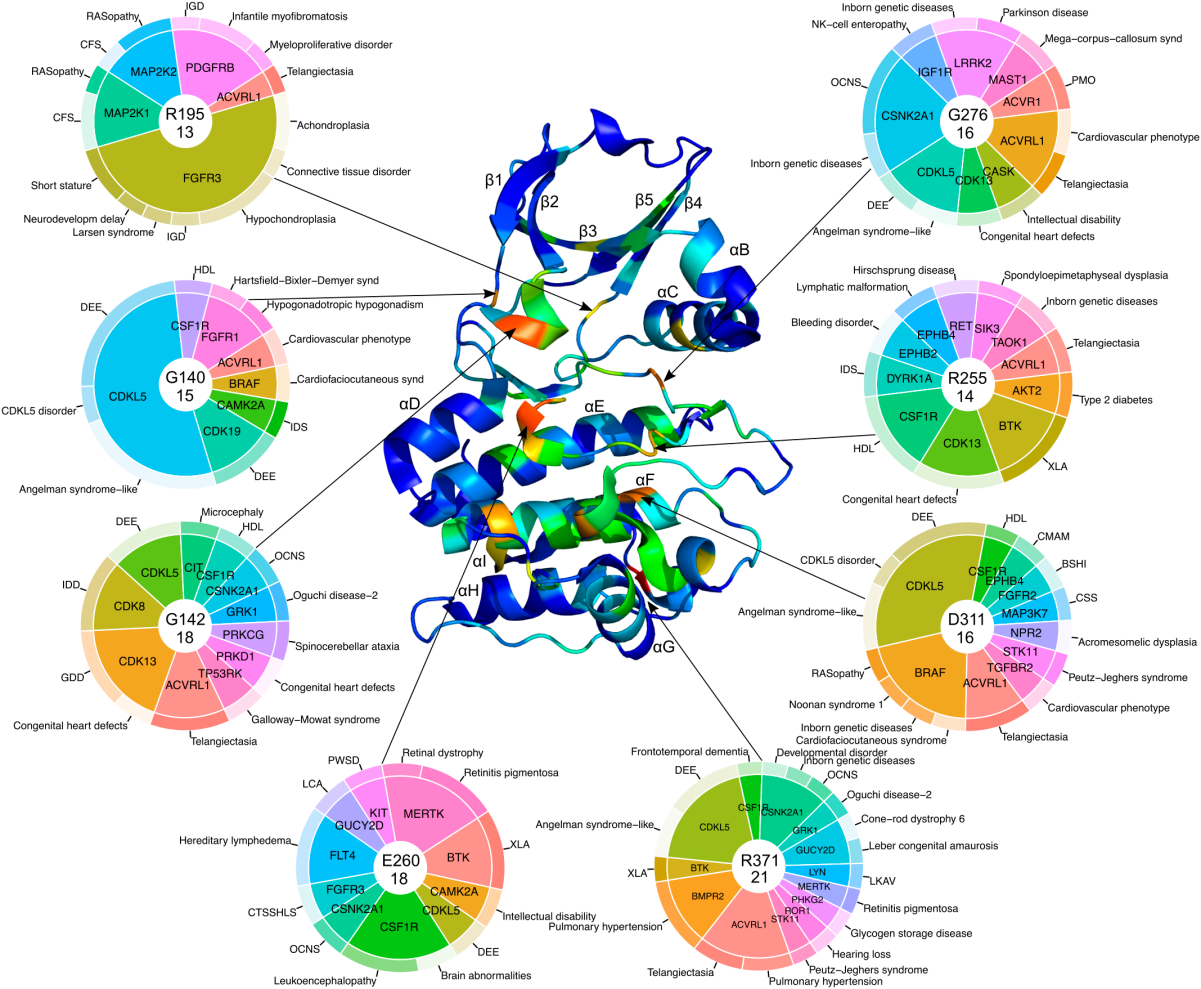
Non‐cancer‐related mutations mapped to kinase domain of Aurora kinase A (PDB: 3E5A). Pie charts represent genes (inner layer) and medical conditions and diseases (outer layer) that are related to mutations in a particular position. One mutation can be related to several diseases. In the center of each pie chart the top item represents the number of amino acid in 3E5A structure, the bottom item represents the total number of SNVs in this position in MSA. BSHI, bilateral sensorineural hearing impairment; CFS, cardiofaciocutaneous syndrome; CMAM, capillary malformation‐arteriovenous malformation; CSS, cardiospondylocarpofacial syndrome; CTSSHLS, camptodactyly‐tall stature‐scoliosis‐hearing loss syndrome; DEE, developmental and epileptic encephalopathy; GDD, global developmental delay; HDL, hereditary diffuse leukoencephalopathy; IDD, intellectual developmental disorder; IDS, intellectual disability syndrome; IGD, inborn genetic diseases; LCA, Leber congenital amaurosis; LKAV, LYN kinase‐associated vasculopathy; OCNS, Okur‐Chung neurodevelopmental syndrome; PMO, progressive myositis ossificans; PWSD, piebaldism with sensorineural deafness; XLA, X‐linked agammaglobulinemia.

We mapped all pathogenic mutations in 497 human protein kinase domains (Modi & Dunbrack Jr., 2019) from ClinVar database (Landrum et al., 2020) to the reference structure of Aurora kinase A (AURKA) and grouped them by the relevance to the disease type (cancer‐related and others). Cancer‐related mutations mapped to the Aurora kinase A 3D structure revealed eight major hotspots (Figure 1). Most of the hotspots are located within catalytic and activation loops of kinase domain (Dixit et al., 2009). The hotspot with the highest number of mutations (20) corresponds to H280 of AURKA, located in the activation loop. This hotspot includes mutations from six kinase genes with the BRAF gene being responsible for the largest number of various cancer types (Figure 1). BRAF V600 is a known driver mutation that is related to poor prognosis in colorectal cancer (Chen et al., 2014), lung cancer (Cardarella et al., 2013), melanoma (Flaherty et al., 2012), and glioblastoma (Chang et al., 2016). Driver mutations from other kinases localized in this hotspot include EGFR L861 (lung cancer [Mitsudomi & Yatabe, 2010]), FLT3 D835 (leukemia [Martelli et al., 2013]), KIT D816 (leukemia [Smith & Shah, 2013], melanoma [Willmore‐Payne et al., 2006]), MET D1228 (carcinoma [Lorenzato et al., 2002]), PDGFRA D842 (gastrointestinal tumors [Heinrich et al., 2012]). Two cancer‐related hotspots with 11 total mutations correspond to R195 and D274 of the AURKA structure (Figure 1). R195 includes mutations in five genes: FGFR2 (endometrial carcinoma [Dutt et al., 2008], breast neoplasm [Chang et al., 2016]), FLT3 (leukemia [Zhang et al., 2014]), KIT (gastrointestinal stromal tumors [Fornasarig et al., 2020]), MAP2K1 (melanoma [Emery et al., 2009]), and PDGFRA (gastrointestinal stromal tumors [Corless et al., 2005]). R195 also includes 13 non‐cancer‐related mutations in five genes (Figure 2): FGFR3 (hypochondroplasia [Xue et al., 2014]), PDGFRB (myofibromatosis [Arts et al., 2016]), MAP2K1 and MAP2K2 (cardio‐facio‐cutaneous syndrome [Schulz et al., 2008]), and ACVRL1 (telangiectasia [Koenighofer et al., 2019]). D274 locates in activation loop of kinase domain and includes mutations from two genes: BRAF (melanoma [Maldonado et al., 2003]) and STK11 (lung cancer [Ji et al., 2007]). Two cancer‐related hotspots with 10 mutations correspond to V252 (catalytic loop) and A281 (activation loop) (Figure 1). V252 includes mutations in two genes ALK (neuroblastoma [Schonherr et al., 2011]) and ABL1 (chronic myelogenous leukemia [Cho et al., 2013]). A281 includes mutations in three genes: BRAF (melanoma [Chang et al., 2016]), FLT3(leukemia [Mills et al., 2006]), and MAP2K1 (neoplasms [Morris et al., 2013]). The final cancer‐related hotspot located within the activation loop is G276 which has 7 mutations in three genes: BRAF (glioblastoma [Chang et al., 2016]), STK11 (non‐small cell lung carcinoma [MacConaill et al., 2014]), and CDK12 (lung adenocarcinoma [Biswas et al., 2016]).

In the contrast to the cancer‐related mutations, non‐cancer‐related hotspots are mostly located outside of catalytic and activation loops of kinase domain and include larger number of involved genes (Figure 2). The hotspot with the highest number of exclusively non‐cancer‐related pathogenic mutations (21) corresponds to R371 of the AURKA structure and is located at the C‐terminal part of αH of the kinase domain. To the best of our knowledge, this hotspot has not been described previously. R371 includes mutations in 15 genes that correspond to seven kinase groups (Table 1). The majority of these kinase groups (five out of seven) are Ser/Thr protein kinases (AGC, CAMK, CMGC, STE, TKL). The rest are Receptor Guanylate Cyclase (RGC) and Tyr protein kinases (TYR). Tyrosine kinases are the prevalent group among proteins with mutations in the R371 hotspot (6 out of 15). Based on their Medical Subject Heading (MeSH) disease classification (Rogers, 1963) the majority of diseases caused by mutations from these hotspots belong to classes of nervous system diseases, such as encephalopathy (Hector et al., 2017), Okur‐Chung syndrome (Owen et al., 2018), and X‐linked agammaglobulinemia (Lopez‐Herrera et al., 2008); as well as eye diseases such as Leber congenital amaurosis (Thompson et al., 2017). Enrichment analysis of 15 kinases related to the R371 hotspot using ShinyGO (Ge et al., 2020) revealed enrichment of these proteins in the NF‐kappa B signaling pathway, which is distinctive when using the whole human proteome as a background (Enrichment FDR = 2.3E‐05, Fold Enrichment = 62.6) and our dataset of kinases (Enrichment FDR = 4.6E‐02, Fold Enrichment = 8.2). The NF‐kappa B signaling pathway is known for its key role in inflammatory processes (Liu et al., 2017); however, it also plays an important role in the function of the nervous system (Dresselhaus & Meffert, 2019; Kaltschmidt & Kaltschmidt, 2009) and the regulation of ocular surface inflammation (Lan et al., 2012).

**TABLE 1:** Disease classes that are caused by non‐cancerous mutations corresponded to R371 hotspot of Aurora kinase A.

Gene name	UniProt ID	Kinase group	Disease	MeSH class
GRK1	Q15835	AGC	Oguchi disease	Eye diseases
PHKG2	P15735	CAMK	Glycogen storage disease IXC	Nutritional and metabolic diseases
STK11	Q15831	CAMK	Peutz‐Jeghers syndrome	Digestive system diseases; skin and connective tissue diseases
CDKL5	O76039	CMGC	Developmental and epileptic encephalopathy	Nervous system diseases
			Angelman syndrome‐like	Nervous system diseases
CSNK2A1	P68400	CMGC	Okur‐Chung neurodevelopmental syndrome	Nervous system diseases
GUCY2D	Q02846	RGC	Cone‐rod dystrophy 6	Eye diseases
			Leber congenital amaurosis 1	Eye diseases
TAOK1	Q7L7X3	STE	n/a	n/a
ACVRL1	P37023	TKL	Pulmonary hypertension	Respiratory tract diseases
			Telangiectasia, hereditary hemorrhagic	Hemic and lymphatic diseases; cardiovascular diseases
BMPR2	Q13873	TKL	Pulmonary hypertension	Respiratory tract diseases
BTK	Q06187	TYR	X‐linked agammaglobulinemia with growth hormone deficiency	Immune system diseases; nervous system diseases; endocrine system diseases
CSF1R	P07333	TYR	Frontotemporal dementia	Nervous system diseases; mental disorders
LYN	P07948	TYR	LYN kinase associated vasculopathy	Diseases of the musculoskeletal system and connective tissue
MERTK	Q12866	TYR	Retinitis pigmentosa 38	Eye diseases
ROR1	Q01973	TYR	Hearing loss, autosomal recessive 108	Nervous system diseases; otorhinolaryngologic diseases
ZAP70	P43403	TYR	n/a	n/a

Abbreviation: MeSH, Medical Subject Headings.

Two non‐cancer‐related hotspots with 18 mutations correspond to G142 and E260. G142 is located within the Gly‐rich loop of the N‐terminal lobe of the kinase domain and includes non‐cancer‐related mutations in 11 genes such as: CIT (microcephaly [Li et al., 2016]), CDK8 (intellectual developmental disorder [Calpena et al., 2019]), TP53RK (Galloway‐Mowat syndrome [Braun et al., 2017]). This hotspot also includes four cancer‐related mutations in two genes (Figure 1): BRAF (non‐small cell lung carcinoma [Houben et al., 2004]) and FLT3 (acute myeloid leukemia [Zhang et al., 2014]). E260 locates within catalytic loop and includes non‐cancer‐related mutations in 10 genes, for example: BTK (agammaglobulinemia [Sigmon et al., 2008]), CSF1R (leukoencephalopathy [Kinoshita et al., 2012]), FGFR3 (hearing loss syndrome [Toydemir et al., 2006]). The hotspot D311 locates in the N‐terminal part of αF and includes 16 mutations in 10 genes such as: CDKL5 (encephalopathy [Melikishvili et al., 2019]), ACVRL1 (telangiectasia [Letteboer et al., 2005]), MAP3K7 (cardiospondylocarpofacial syndrome [Le Goff et al., 2016]).

Another hotspot with 16 mutations corresponds to G276 of the AURKA structure. It is the only non‐cancer‐related hotspot located within the activation loop and includes mutations in 9 genes such as MAST1 (mega‐corpus‐callosum syndrome [Tripathy et al., 2018]), ACVRL1 (telangiectasia [Schulte et al., 2005]). This hotspot also includes the seven cancer‐related mutations discussed above (Figure 1). Hotspot G140 is located in the Gly‐rich loop region of kinase domains and includes 15 non‐cancer‐related pathogenic mutations in seven genes, for example: CDK19 (encephalopathy [Zarate et al., 2021]), CSF1R (leukoencephalopathy [Rademakers et al., 2011]). This hotspot also includes seven cancer‐related mutations in two genes (Figure 1): BRAF (melanoma [Carlino et al., 2014]) and EGFR (non‐small cell lung carcinoma [Berge et al., 2013]). Finally, R255 is located within catalytic loop and includes non‐cancer‐related mutations in 11 genes such as AKT2 (type 2 diabetes [George et al., 2004]), DYRK1A (intellectual disability [Ji et al., 2015]), and CSF1R (leukoencephalopathy [Guerreiro et al., 2013]).

A previous pan‐cancer study revealed that among protein domains significantly enriched in mutations, the protein kinase domain (Pfam ID: PF07714) is the second most mutated, considering all detected mutations (Miller et al., 2015). The top three kinase genes with the largest number of mutations in pan‐cancer dataset included BRAF, EGFR and ERBB2 (Miller et al., 2015). Our analysis of cancer‐related pathogenic mutations from the ClinVar database showed that the top three genes (containing the most pathogenic cancer‐related mutations) are BRAF, ALK, ABL1, and EGFR and ERBB2 are in the top 11 (Table 2).

**TABLE 2:** Top kinase genes with largest number of pathogenic cancer‐related mutations.

Gene name	UniProt ID	Number of mutations
BRAF	P15056	53
ALK	Q9UM73	27
ABL1	P00519	26
FLT3	P36888	26
STK11	Q15831	21
EGFR	P00533	19
MAP2K1	Q02750	18
KIT	P10721	18
FGFR2	P21802	17
MET	P08581	17
ERBB2	P04626	14

**FIGURE 3:**
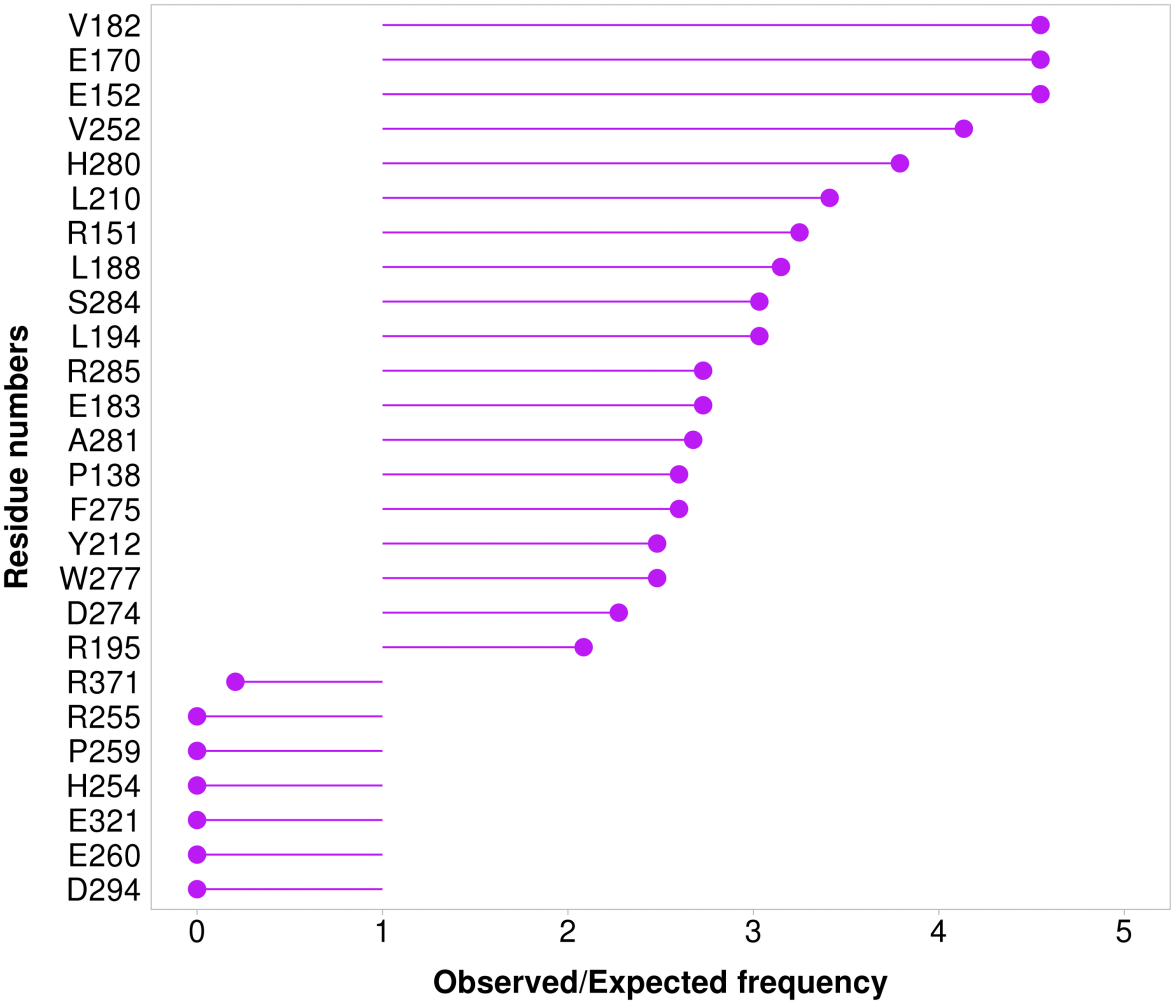
The ratio of observed and expected frequencies of cancer‐related mutations in specific positions of Aurora kinase A (*p*‐value < 0.05). Ratio >1 defines overrepresentation of cancer‐related and underrepresentation of non‐cancer‐related mutations in a position. Ratio <1 defines underrepresentation of cancer‐related and overrepresentation of non‐cancer‐related mutations in a position.

**FIGURE 4:**
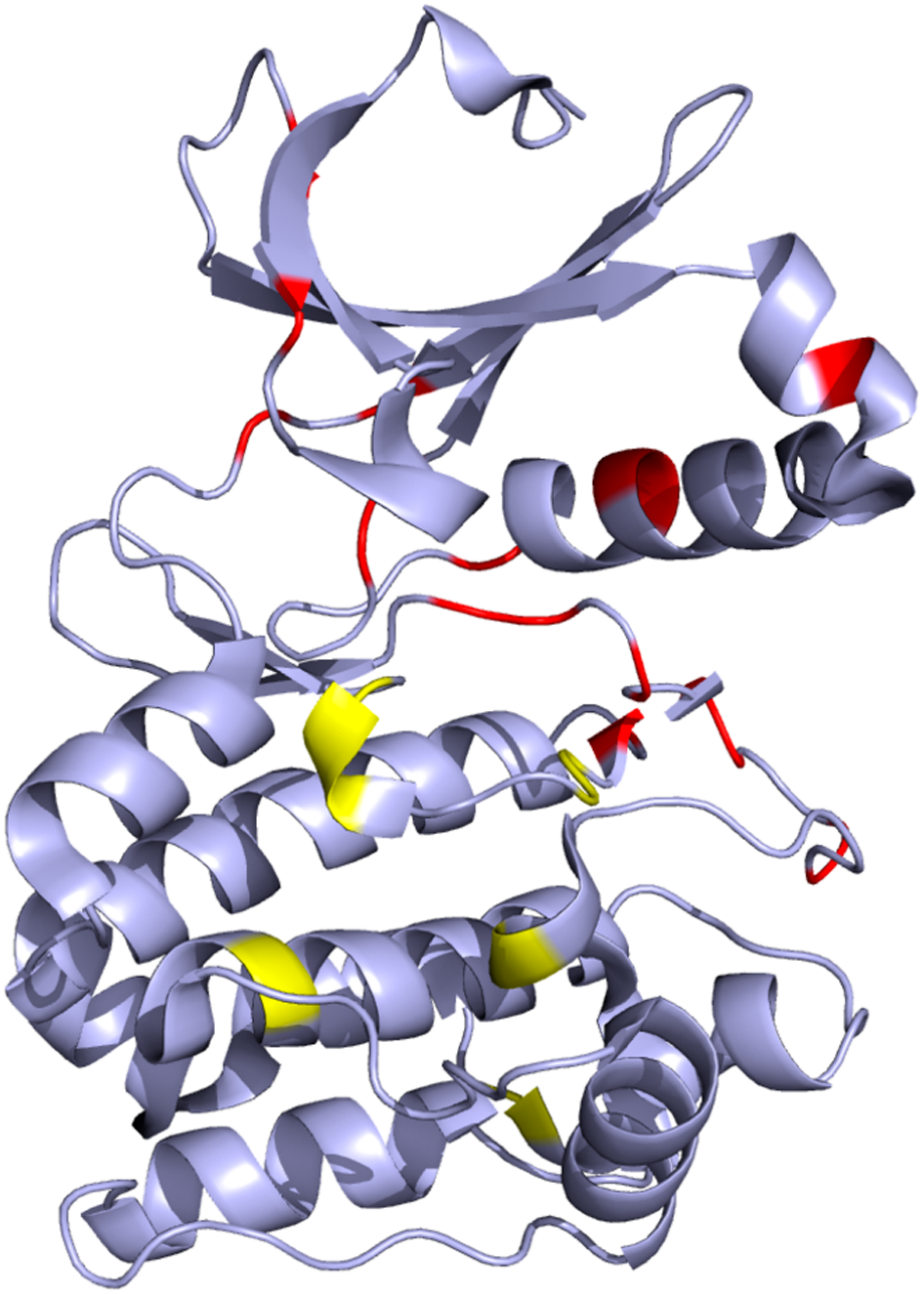
Positions of Aurora kinase A with an overrepresentation of cancer‐related (red) and non‐cancer‐related (yellow) mutations.

Furthermore, we compared the statistics of cancerous and non‐cancerous mutations by calculating the over and underrepresentation of these mutations at each position (see Materials and Methods). Our analysis revealed 19 positions with significant (*p*‐value <0.05) overrepresentation of cancerous mutations and seven positions with significant overrepresentation of non‐cancerous mutations (Figure 3, Table S1). Mapping these positions to the Aurora kinase A structure revealed that positions with an overrepresentation of cancerous mutations are predominantly located in the N‐terminal region of the kinase domain and within the catalytic loop (Figure 4). Only one position with overrepresented cancer‐related mutations is located within the activation loop. On the other hand, positions with overrepresentation of non‐cancerous mutations are mostly located in C‐terminal region of the domain and within the activation loop (Figure 4).

We investigated the intrinsic tendency of each residue to mutate by collecting all missense mutations with benign clinical relevance and a population frequency greater than or equal to 1% from the gnomAD database (Gudmundsson et al., 2022) for all kinases in our dataset. Overall, we identified 26 such mutations with maximum four mutations per position of Aurora kinase A structure (Table S1). Benign mutations are predominantly located on the surface of the protein structure and outside of the catalytic and activation loops (Figure S1). Moreover, the majority of cancerous and non‐cancerous mutation hotspots do not share positions in common with benign missense mutations (Table S1).

Thus, our analysis revealed major cancer‐related pathogenic mutation hotspots in the structure of the kinase domain located mostly within the catalytic and activation loops. Non‐cancer‐related hotspots are mostly located outside of catalytic and activation loops of kinase domain and include a larger number of involved genes. Protein kinases play dual roles as tumor suppressors and proto‐oncogenes in normal, healthy cells. As a consequence, mutations in protein kinases often promote a diverse array of tumorigenic activities. Due to their crucial involvement in DNA damage response and cell cycle checkpoints, protein kinases are associated with numerous loss‐of‐function mutations that can lead to the inhibition of apoptosis, the acquisition of a “mutator” phenotype, and eventual tumorigenesis (Torkamani et al., 2009). Structures of protein kinases have unveiled significant structural distinctions between the closely related active and highly specific inactive forms of kinases (Nolen et al., 2004). For instance, the conformational landscape of ABL kinase, which encompasses active, inactive, intermediate, and inactive‐like conformations, has provided evidence that the various structures of the kinase activation loop may indeed reflect natural kinase conformations (Cowan‐Jacob et al., 2005). Hence, it is likely that activating mutations disturb this equilibrium, favoring the active conformation by either destabilizing the inactive state or stabilizing the active state of the protein kinase. This mechanism could potentially contribute to certain mutations' ability to promote tumorigenesis and could be the reason for the locations of cancerous mutations within the catalytic and activation loops.

## 3 MATERIALS AND METHODS

### 3.1 Data collection

We used a structure‐based multiple sequence alignment (MSA) of 497 human protein kinase domains from Modi and Dunbrack Jr. (2019). This MSA does not consider atypical kinases. For each protein in the MSA “Pathogenic” and “Likely Pathogenic” single nucleotide variants (SNVs) that result in single amino acid substitutions were retrieved from ClinVar database (Landrum et al., 2020). Retrieved SNVs were considered as pathogenic and were mapped to MSA and to the reference structure of Aurora kinase A (Gene name: AURKA, UniProt: O14965, PDB: 3E5A [Zhao et al., 2008]). The reference structure was selected based on previous study (Modi & Dunbrack Jr., 2019). Total numbers of SNVs were summarized for each amino acid position in AURKA and were treated as B‐factors in the PDB file for corresponding C‐alpha atoms. The resulting PDB file was visualized using PyMOL. SNVs located in the gap regions of AURKA in MSA were excluded from consideration. In this study, we determined hotspot mutations by counting the frequency of their occurrence in kinase domains. The top 5% positions with the highest frequency of mutation for cancer‐related and non‐cancer‐related cases were considered as hotspots. Medical conditions and diseases that are related to SNVs in human protein kinase domains were retrieved from ClinVar database. Cancer‐related medical conditions were sorted out manually from the total list. Pie charts were created using R package, v4.2.1, library *webr* and *PieDonut* function with following settings for *PieDonut* arguments: showRatioPie = FALSE, showRatioDonut = FALSE, labelpositionThreshold = 0.5, donutLabelSize = 3.0, pieLabelSize = 3.5, r0 = 0.2, r1 = 0.7, r2 = 0.8, maxx = 1.7, start = 1.

### 3.2 Statistical analysis

Comparison of the number of cancerous and non‐cancerous mutations for each position was conducted using over and underrepresentation, which was calculated as ratio of observed and expected frequencies. The observed frequency for cancer mutations in each Aurora kinase A position was calculated as a ratio of the number of the cancerous mutations in this position over the sum of all cancerous mutations mapped to Aurora kinase A structure. The observed frequency for non‐cancer mutations in each Aurora kinase A position was calculated as a ratio of the number of the non‐cancerous mutations in this position over the sum of all non‐cancerous mutations mapped to Aurora kinase A structure. The expected frequency for each position was calculated as ratio of total number of mutation (cancerous and non‐cancerous) in this position of Aurora kinase A over the total number of mutations. Significance of over and underrepresentation was checked using chi‐square test (*p*‐value<0.05 is considered significant). Statistical analysis was conducted using the R package, v4.2.1.

### 3.3 Analysis of benign mutations

To examine the inherent propensity of each residue to mutate, we obtained all missense mutations with benign clinical relevance and frequency in population greater than or equal to 1% from gnomAD v2.1.1 database (Gudmundsson et al., 2022) for all human protein kinases in our dataset. Obtained benign mutations were mapped to human kinases MSA and to the reference structure of Aurora kinase A (Figure S1).

## AUTHOR CONTRIBUTIONS

**Kirill E. Medvedev**: Conceptualization; methodology; software; validation; formal analysis; investigation; data curation; visualization; writing—original draft; project administration. **R. Dustin Schaeffer**: Writing—review and editing; funding acquisition. **Jimin Pei**: Writing—review and editing. **Nick V. Grishin**: Conceptualization; resources; funding acquisition; writing—review and editing.

## CONFLICT OF INTEREST STATEMENT

The authors declare that there are no competing interests associated with the manuscript.

## Supporting information


**Figure S1.** Missense mutations with benign clinical relevance mapped to kinase domain of Aurora kinase A (PDB: 3E5A). Color shows mutations frequency—from minimum (blue) to maximum (red).

**Table S1.** Statistics for cancer and non‐cancer mutations mapped to Aurora kinase A structure.
