# Supplementary material for: Pathogenic mutation hotspots in protein kinase domain structure
Source: Protein Sci. 2023 Sep 1;32(9):e4750. doi: 10.1002/pro.4750 (PMC10464295; doi:10.1002/pro.4750)
Supplement: Supplementary file 1 — Figure S1. Missense mutations with benign clinical relevance mapped to kinase domain of Aurora kinase A (PDB: 3E5A). Color shows mutations frequency—from minimum (blue) to maximum (red). [file PRO-32-e4750-s002.pdf]

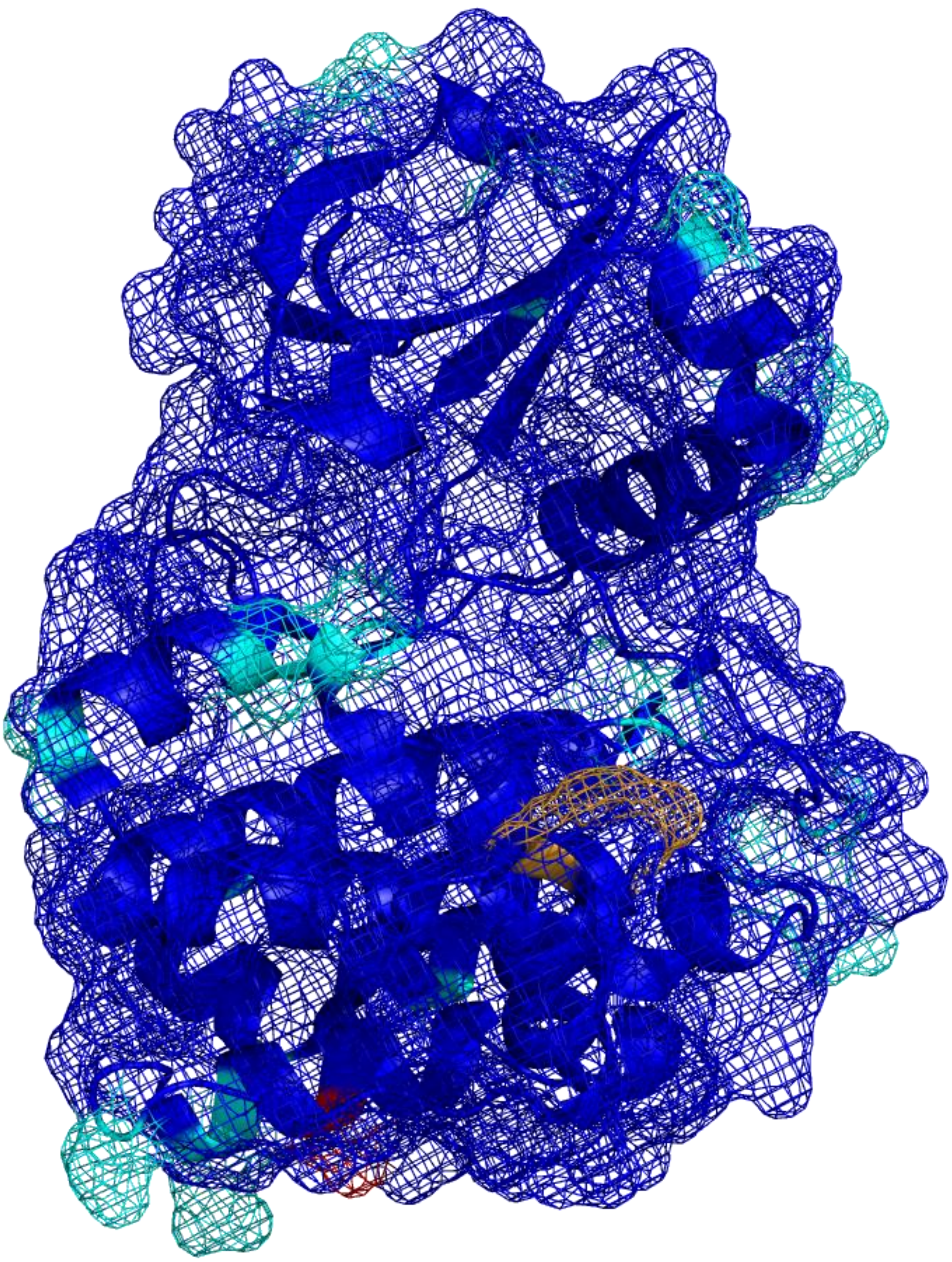

**Figure S1. Missense mutations with benign clinical relevance mapped to kinase domain of Aurora kinase A (PDB: 3E5A).** Color shows mutations frequency – from minimum (blue) to maximum (red).
